# Dynamic Bioreactor Culture for Infiltration of Bone Mesenchymal Stem Cells within Electrospun Nanofibrous Scaffolds for Annulus Fibrosus Repair

**DOI:** 10.1111/os.12615

**Published:** 2020-01-16

**Authors:** Shuang Wang, Yun‐Fei He, Jun Ma, Lei Yu, Jian‐Kun Wen, Xiao‐Jian Ye

**Affiliations:** ^1^ Department of Spinal Surgery, Changzheng Hospital Second Military Medical University Shanghai China; ^2^ Department of Orthopedics The 940th Hospital of Joint Logistics Support Force of the Chinese People's Liberation Army Lanzhou China; ^3^ Department of Orthopedics Central Theater Command General Hospital of the Chinese People's Liberation Army Wuhan China

**Keywords:** Annulus fibrosus, Cell culture techniques, Mesenchymal stem cells, Tissue engineering, Tissue scaffolds

## Abstract

**Objective:**

To compare the ability of three culture strategies of static culture, intermittent centrifugal culture and dynamic bioreactor culture in promoting the infiltration of bone marrow mesenchymal stem cells (BMSCs) throughout electrospun nanoporous aligned nanoyarn scaffold (AYS).

**Methods:**

AYS was constructed by the method of conjugated electrospinning, using the blended solution of poly (L‐lactide‐co‐caprolactone) (P (LLA‐CL)) and gelatin. Then the bone marrow mesenchymal stem cells (BMSCs) were transplanted on the scaffolds. Culture the scaffold‐cells using three methods of static culture, intermittent centrifugal culture and dynamic bioreactor culture. After 7 and 14 days in culture, the infiltration depth of the cells were observed and measured by hematoxylin and eosin (HE) or 4′, 6‐diamidino‐2‐phenylindole (DAPI) staining.

**Result:**

In the current study, on the 7th day, the BMSCs in the scaffolds of static culture group, intermittent centrifugal culture group, and dynamic bioreactor culture group infiltrated to an average depth of 11.88 ± 1.82 μm, 21.17 ± 13.17 μm, and 26.27 ± 7.42 μm, respectively. There were differences between the bioreactor culture group with the static culture group and the intermittent centrifugal culture group. On the time point of 14 days, the depth of infiltration of BMSCs in dynamic bioreactor culture was the most (115.13 ± 25.44 μm, *P* < 0.05), and the infiltration of the cells in the intermittent centrifugal culture group was 42.53 ± 13.07 μm, deeper than that of the static culture group (24.53 ± 6.06, *P* < 0.05).

**Conclusion:**

Dynamic bioreactor culture may be a preferred method for tissue engineering approaches involving scaffolds with a low porosity, such as those needed for repair of the annulus fibrosus (AF).

## Introduction

Discectomy is a common surgical treatment for patients with intervertebral disc herniation (IDH), and it can relieve symptoms caused by compression of nerve roots by the protruding disc, including pain, numbness, and muscle weakness. For excision of the nucleus pulposus, the annulus fibrosus (AF), which surrounds the nucleus pulposus, must be incised to some extent. Because the AF functions to not only encase the nucleus pulposus but also to maintain pressurization of the intervertebral disc (IVD), degeneration of the IVD will be accelerated^1^ if the AF is not repaired and herniation of the disc may recur^2^, ^3^, ^4^.

Several strategies have been developed for repair of the AF, such as suturing the AF directly^5^ or filling the gap with an appropriate material such as collagen gel^6^. These methods can restore the integrity of the AF, at least partially, but they cannot restore the pressurization of the IVD, and thus, the ability of the IVD to resist a loaded force remains diminished^7^. For these reasons, regeneration of the IVD is not promoted effectively. Recently, tissue engineering strategies have been developed for the immediate repair of the AF with an appropriate scaffold material^8^ containing biologically appropriate cells^9^ for regeneration of the AF^10^.

Various types of materials have been employed in the construction of scaffolds for tissue engineering strategies related to AF repair, such as porous silk fibroin, polycaprolactone (PCL), and fibrin cross‐linked with genipin^11^, ^12^, ^13^, ^14^, and limitations have been reported for all so far. Because the scaffolds must withstand the force applied to the spine, especially during bending, rotating, or twisting, the scaffolds used to repair spinal tissue must have sufficient compressive and tensile strength. Another essential property of the scaffolds is the appropriate porosity for penetration and growth of cells. Because strength and porosity are opposing properties, a delicate balance is required. One approach to reducing the need for a high porosity involves promoting the delivery of cells within the scaffold structure. In the current study, we employed a nanofibrous scaffold (aligned nanoyarn scaffold, AYS) fabricated by electrostatic spinning to mimic the structure of the outer layer of the AF as described previously^15^. These scaffolds have appropriate mechanical properties for the repair of AF. However, in our previous study, we found that it is hard to achieve a uniform distribution of cells throughout these scaffolds, especially in the inner‐most part of the scaffolds. So it is necessary to infiltrate more cells into the scaffolds using appropriate method of culturing.

In tissue engineering, the static culture is a useful strategy and the most commonly technology employed. However, this technology does not have the capacity for promoting the infiltration of the cells cultured on the scaffolds. In order to increase the cells infiltrating into the scaffolds, some researchers applied the method of intermittent centrifugal culture and achieved deeper infiltration of the cells^16^, ^17^, ^18^, ^19^. Meantime, the uneven distribution of the cells was observed, which may be associated with the force produced during the centrifugation. The strategy of dynamic bioreactor culture is to simulate the *in vivo* conditions within three‐dimensional constructs. This method makes the exchange of both nutrients and oxygen more effective in the inner part of the scaffolds by the perfusion flow. Since the dynamic bioreactor culture does not apply the process of centrifugation, it may avoid the disadvantage of the intermittent centrifugal culture.

Therefore, in the present study, we aimed to: (i) culture the bone marrow mesenchymal stem cells (BMSCs) using three culture strategies of static culture, intermittent centrifugal culture, and dynamic bioreactor culture; (ii) measure and compare the depths of the BMSCs infiltrated into the scaffolds; (iii) identify the optimal strategy for promoting the infiltration of BMSCs into the scaffold in tissue engineering.

## Materials and Methods

### Materials

Gelatin was purchased from MP Biomedicals LLC (Irvine, CA, USA) and hexafluoroisopropanol (HFIP) from Darui Co., Ltd. (Shanghai, China). Then poly (L‐lactide‐co‐caprolactone) (P(LLA‐CL)) was provided by GUNZE Co., Ltd. (Kyoto, Japan).

### Scaffold Formation

**Figure 1:**
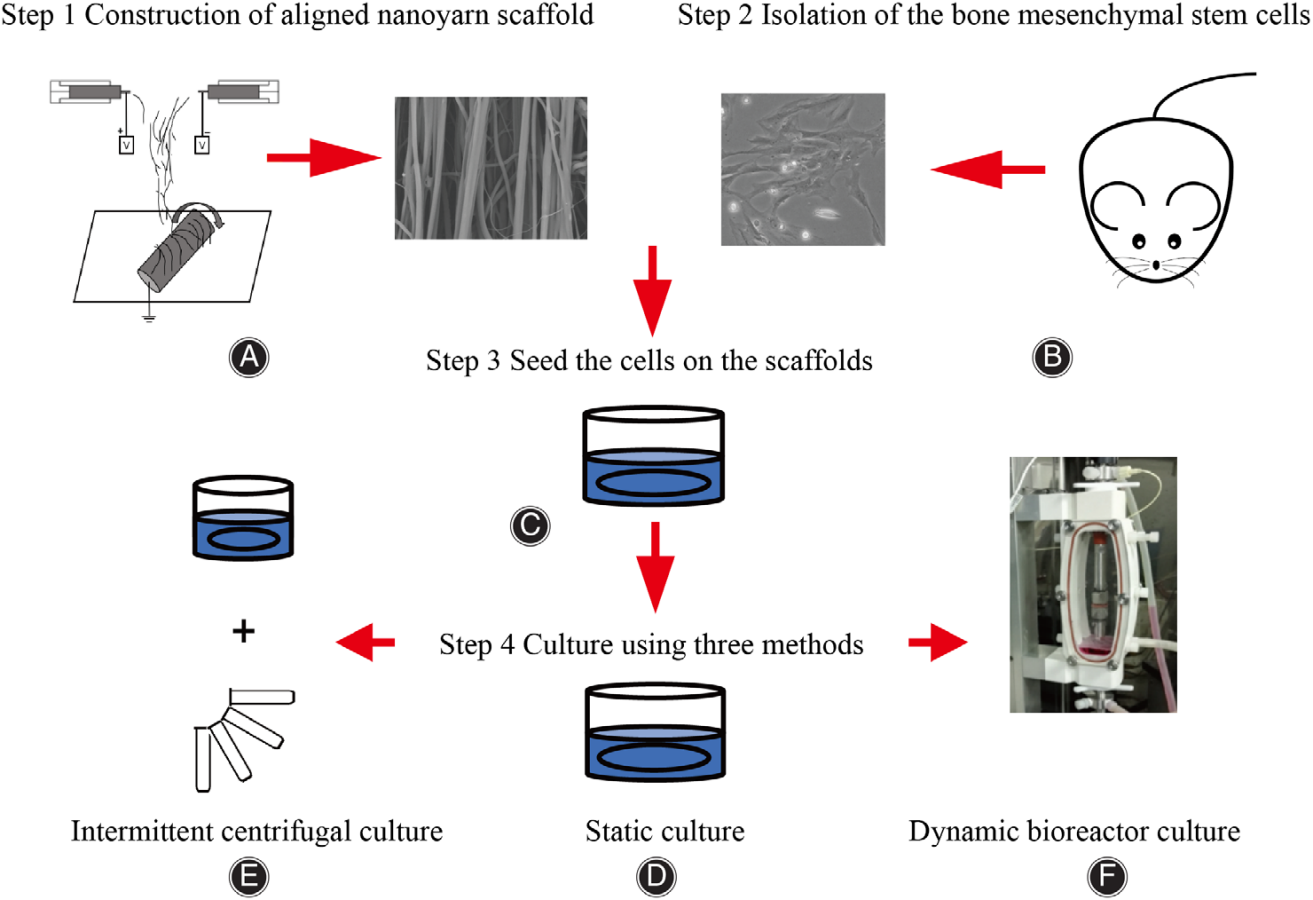
Process schematic diagram of the current study. (A) Conjugated electrospinning were applied to construct the aligned nanoyarn scaffold. The bone marrow mesenchymal stem cells were isolated (B) and seeded on the up surfaces of the scaffolds (C). The cell‐laden scaffolds of the static culture were placed in the 24‐well plates and the medium was changed every 2–3 days (D), while in the intermittent centrifugal culture the process of centrifugated was added every 2–3 days when changing the medium (1000 rpm, 5 min) (C, E). As for the dynamic bioreactor culture, the BioDynamic (BOSE5100; Bose Electroforce, New Castle, PA, USA) was applied to culture the cells after seeding (C, F).

The AYS constructs were prepared by the method of conjugated electrospinning as described previously (Fig. 1A)^15^. Briefly, 10 mL of gelatin was dissolved in 10 mL HFIP at 12% (w/v) and 3 mL of P (LLA‐CL) dissolved in 10 mL HFIP at 8% (w/v) were mixed together. The blended solution was stirred until transparent and poured into two syringes fixed with 9G needles. The syringes were placed at the same height of 15 cm and opposing electrical voltages of 12 kV were applied to the needles respectively. The jet impelling rate was set at 1.5 mL/h. The fibers entangled to form polymer composite nanoyarns as they carried the opposite charges. The nanoyarns were collected continuously by a mandrel with a diameter of 15 cm and rotating as 140 rpm. Nanoyarns were collected until a scaffold formed with a thickness of approximately 250 μm in order to mimic the thickness of the AF^20^. The collected scaffolds were first kept in a vacuum oven for 7 days, then immersed in 75% ethanol for 4 h, and finally washed with phosphate‐buffered saline (PBS) (Thermo Fisher Scientific, Waltham, MA, USA) three times before use.

### Bone Mesenchymal Stem Cells Isolation and Culture

The animal protocols used for the experiments in this study were approved by the Institutional Animal Care and Use Committee of the Second Military Medical University (Shanghai, China). BMSCs were isolated from adult Sprague–Dawley rats with an average weight of 50 g according to a previously published protocol^21^. After sacrifice of the rats by cervical dislocation, the tibias and femurs were harvested in an aseptic manner. The bone marrow cavity was exposed after removal of the adherent soft tissue and the ends of the bone. The marrow was flushed out and suspended in the complete medium composed of Dulbecco's Modified Eagle's Medium (DMEM) (Thermo Fisher Scientific, Waltham, MA, USA), 10% fetal bovine serum (FBS) (Thermo Fisher Scientific, Waltham, MA, USA) and 1% penicillin/streptomycin (Thermo Fisher Scientific, Waltham, MA, USA) for plating in tissue culture flasks. The cells were allowed to adhere for 24 h and non‐adherent cells were removed, then the medium was replaced every 2–3 days. The experiments presented herein were carried using the third passages of BMSCs (Fig. 1B).

### Techniques for Bone Mesenchymal Stem Cells Culture within Electrospun Scaffolds

For culture using the three techniques, round scaffolds approximately 6 mm in diameter and 250 μm in thickness were prepared and placed in the wells of 24‐well plate. Then the culture methods were applied as per the following steps.

### Static Culture

The cells were seeded on the up‐side of the surfaces of the scaffolds and kept in the 24‐well plates submerged in the complete medium. The complexes were cultivated under the condition of 37°C with 5% CO_2_ and the medium was changed every 2–3 days (Fig. 1C,D).

### Intermittent Centrifugal Culture

BMSCs were seeded on the up‐surface of scaffolds and cultured as described in static culture but with centrifugation for 5 min at 1000 rpm (100 × *g*) every 2–3 days when changing the medium. For centrifugation, the scaffolds were moved to centrifuge tubes and then returned to the wells under aseptic conditions (Fig. 1C,E).

### Dynamic Bioreactor Culture

BMSCs were seeded onto the surface of scaffolds as described for the static culture. After that, the complexes were transferred to the BioDynamic (BOSE5100; Bose Electroforce, New Castle, PA, USA) under aseptic conditions. The complexes were placed randomly in the bioreactor and cultured using the same medium as in static culture and at 37°C with 5% CO_2_ (Fig. 1C,F).

### Scaffold and Cell Morphology Observed by Scanning Electron Microscope

Scanning electron microscope (SEM; JSM‐5600, JEOL, Tokyo, Japan) was used to observe the morphology of the AYS before the planting BMSCs and after 7 days of static culture. For observation of the surface morphology, the AYS were sprayed gold and examined under the acceleration voltage of 10kV. After 7 days of static cultivation, the scaffolds with BMSCs were rinsed with PBS twice before fixed in 2.5% glutaraldehyde (Sinopharm Chemical Reagent Co., Ltd., Shanghai, China) for 3 h. Then different concentrations of ethanol (60%, 70%, 80%, 90%, 100%) were applied to dehydrate the scaffolds for 15 min each. After that, the surface of the scaffolds were sputter coated with gold and observed.

### Bone Mesenchymal Stem Cells Adhesion and Proliferation

Cell Counting Kit‐8 (CCK‐8; Dojindo, Kumamoto, Japan) was applied to measure the cell metabolic activity in order to reflect the cell viability. Before measurement, the medium of the wells was removed and the scaffolds were washed with PBS. Then culture medium with CCK‐8 reagent (10%, v) was added into the wells. After 2 h, 100 μL solution was taken from each well and transferred to the 96‐well plate. Use a microplate reader (BioTek, Winooski, VT, USA) to measure the absorbance at 450 nm and recorded the optical density (OD) (n = 5).

The OD values of different groups on the 1^st^ day were compared to reflect the adhesion of the cells. And this measurement was taken before the centrifugation of the group of intermittent centrifugal culture. On the 7th and 14th day, the OD values were compared to compare the proliferation of the BMSCs in different groups (n = 5).

### Histological Analysis

On days 7 and 14 after the seeding of BMSCs onto the scaffolds, BMSCs within the scaffolds were fixed by incubation in 4% paraformaldehyde overnight at 4°C. Following subsequent dehydration, clarification, infiltration and paraffin embedding, 5‐μm‐thick cross‐sections of cell‐laden scaffolds cultured under the different strategies were obtained. The sections were processed for hematoxylin and eosin (H&E) staining (Sigma‐Aldrich Co. LLC, St. Louis, MO, USA) and then observed and photographed using a light microscope (DM4000 B; Leica Microsystems, Wetzlar, Hesse, Germany).

### Evaluation of the Infiltration of Bone Mesenchymal Stem Cells within the Scaffolds

**Figure 2:**
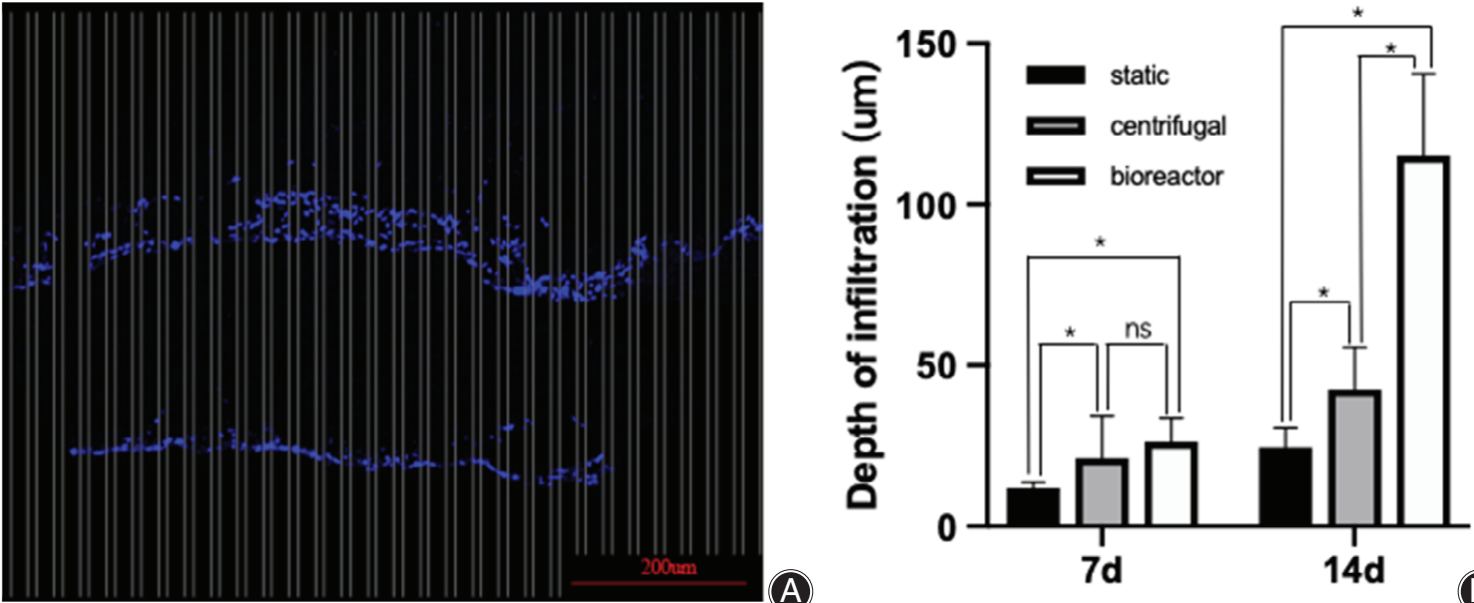
(A) Schematic diagram of the measurement method of the bone mesenchymal stem cells infiltration in the scaffolds. After stained with 4′,6‐diamidino‐2‐phenylindole, the sections of the cell‐laden scaffolds were divided with columns of 10 μm widths at 20 μm intervals. The deepest distances of every column were measured and recorded. (B) Comparison of the BMSCs infiltrating depth in the three groups on the time point of the 7th and the 14th day.

On days 7 and 14 after the seeding of BMSCs onto the scaffolds, cell‐laden scaffolds were collected and flash frozen in liquid N_2_. After embedded in optimal cutting temperature compound (Tissue‐Tek; Sakura Finetechnical Co. Ltd., Tokyo, Japan), the samples were cut into the sections of 10 μm and mounted onto poly(L‐lysine) precoated microscope slides. For fixation, the sections were fixed in 4% polyformaldehyde (Thermo Fisher Scientific, Waltham, MA, USA) for 30 min at room temperature. Then stained the sections with 4′,6‐diamidino‐2‐phenylindole (DAPI; Thermo Fisher Scientific, Waltham, MA, USA). Using the modified method described by Pham *et al*.^22^ (Fig. 2), columns of 10 μm widths were arranged at 20 μm intervals on the sections. The deepest distances of every column were measured and recorded. At least three sections of every sample and three samples of every time point were analyzed to evaluate the infiltration depth data.

### Statistical Analysis

Data are expressed as mean ± standard deviation (SD). Single factor one‐way analysis of variance (ANOVA) was used to compare the infiltrating depth of the cells in different groups when the data are normally distributed, and the intergroup differences were analyzed with the LSD‐t test. Non‐normally distributed data were compared using nonparametric Kruskal‐Wallis test. *P* < 0.05 was considered significant and the statistical analyses were performed using SPSS version 19.0 (IBM Corporation, Armonk, NY, USA). All experiments were repeated at least three times.

## Results

### Morphology of Aligned Nanoyarn Scaffold and Bone Mesenchymal Stem Cells Cultured on the Scaffold

**Figure 3:**
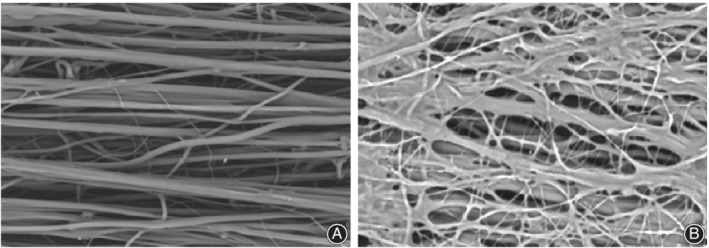
(A) Scanning electron microscope images of the morphology of aligned nanoyarn scaffold. There are two kinds of fibers as aligned nanoyarns and nanofibers composed the three‐dimensional structure of the scaffold. (B) Bone mesenchymal stem cells cultured on the AYS after 7 days. BMSCs were arranged and extended along the nanoyarn fibers.

Observed by SEM before planting the cells, AYS were mainly constructed by the aligned nanoyarns and nanofibers were distributed among them. The two kinds of fibers composed the three‐dimensional structure of the scaffold (Fig. 3A). Then after 7 days of cultivation, BMSCs were observed using SEM (Fig. 3B). BMSCs were arranged and extended along the nanoyarn fibers.

### Adhesion and Proliferation of Bone Mesenchymal Stem Cells on Aligned Nanoyarn Scaffold

**Figure 4:**
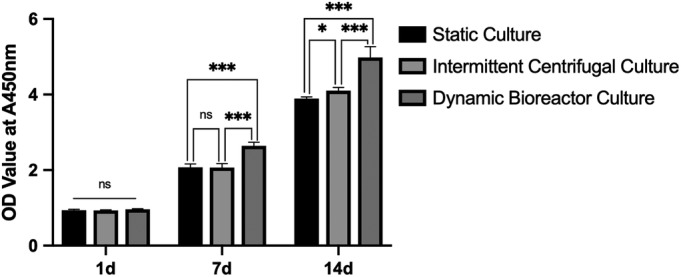
Comparison of the optical density values of three culture methods on the time point of the 1, 7, 14th day. The OD values were similar on the first day. After 7 days, the OD value of the dynamic bioreactor culture was 2.642 ± 0.096, higher than the static culture (2.071 ± 0.093, *P* < 0.05) and the intermittent centrifugal culture (2.067 ± 0.105, *P* < 0.05). On the 14th day, the OD value of the dynamic bioreactor culture was 4.983 ± 0.148, higher than the intermittent centrifugal culture (4.103 ± 0.085, *P* < 0.05) and the static culture (3.892 ± 0.046, *P* < 0.05).

At the first day, the OD values of different groups were similar, which indicating that the cells of different groups have adhered to the surfaces of the scaffolds with nearly the same ability. Then after 7 days of cultivation, the OD value of the dynamic bioreactor culture was 2.642 ± 0.096, higher than the static culture (2.071 ± 0.093, *P* < 0.05) and the intermittent centrifugal culture (2.067 ± 0.105, *P* < 0.05), which indicated that the ability of BMSCs proliferation of the dynamic bioreactor culture group was nearly 30% higher than the other two groups. Meanwhile the OD values of the static culture (2.071 ± 0.093) were similar with the intermittent centrifugal culture (2.067 ± 0.105). On the 14th day, the OD value of the dynamic bioreactor culture was 4.983 ± 0.148, higher than the intermittent centrifugal culture (4.103 ± 0.085, *P* < 0.05) and nearly 25% higher than the static culture (3.892 ± 0.046, *P* < 0.05) (Fig. 4).

### Histological Analysis

**Figure 5:**
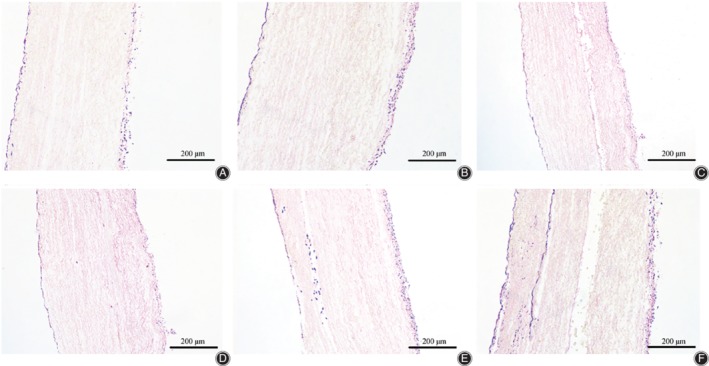
Images of H&E‐stained bone mesenchymal stem cells on and within aligned nanoyarn scaffold after static culture for 7 days (A) or 14 days (D), intermittent centrifugal culture for 7 days (B) or 14 days (E), or dynamic bioreactor culture for 7 days (C) or 14 days (F). After 7 days in culture, cells were observed on the surfaces of all scaffolds with the greatest densities on the static culture (A) or intermittent centrifugal culture (B). After 14 days, the static culture still showed the greatest cells on the surface (D), while many cells were observed in the interior of the intermittent centrifugal culture (E) and dynamic bioreactor culture (F).

After 7 days in culture, BMSCs were observed on the surfaces of all scaffolds (Fig. 5A–C), with the greatest cell densities observed on those subjected to static culture or intermittent centrifugal culture. After 14 days in culture, the cell‐scaffold constructs kept in static culture showed the greatest cells staining intensity on the scaffold surface, with only limited numbers of cells observed in the interior region of the scaffolds (Fig. 5D). By contrast, many cells were observed in the interior of the scaffolds from the intermittent centrifugal culture and dynamic bioreactor culture groups. The intensities of cellular staining on both the surface and within the interior region of the scaffolds appeared greater for the dynamic bioreactor culture group than for the intermittent centrifugal culture group. Moreover, the distribution of cells appeared more uniform within the scaffolds of the dynamic bioreactor culture group than within those of the intermittent centrifugal culture group (Fig. 5D–F).

### Infiltration of Bone Mesenchymal Stem Cells into the Scaffolds

**Figure 6:**
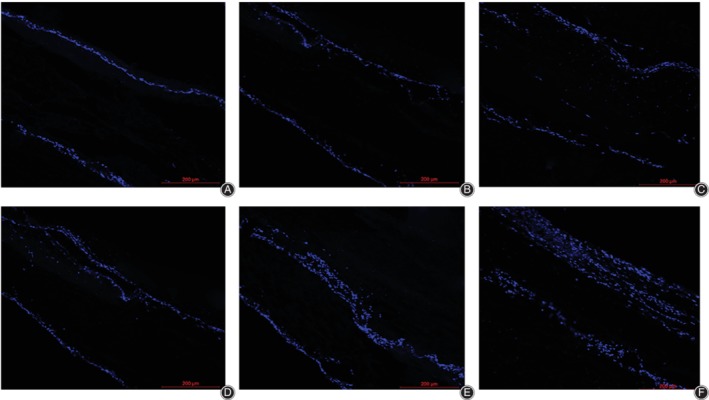
Images of 4′,6‐diamidino‐2‐phenylindole‐stained bone mesenchymal stem cells on and within the scaffolds culture for 7 days (A‐C) or 14 days (D‐F). On the 7th day, the cells in static culture group, intermittent centrifugal culture group, and dynamic bioreactor culture group infiltrated into the scaffolds as the depth of 11.88 ± 1.82 μm, 21.17 ± 13.17 μm, and 26.27 ± 7.42 μm respectively (A‐C). On the 14th day, the depth of BMSCs infiltrated into the scaffolds were 24.53 ± 6.06 μm, 42.53 ± 13.07 μm, and 115.13 ± 25.44 μm. (D‐F) The infiltration depth of the intermittent centrifugal culture was nearly double as the static culture. Meanwhile, the infiltration depth of the dynamic bioreactor culture was as nearly three times as the infiltration depth of the intermittent centrifugal culture.

On day 7, the BMSCs in the scaffolds of static culture group, intermittent centrifugal culture group, and dynamic bioreactor culture group infiltrated the scaffolds to an average depth of 11.88 ± 1.82 μm, 21.17 ± 13.17 μm, and 26.27 ± 7.42 μm, respectively (Fig. 6A–C). These results revealed that the infiltration depth of BMSCs of intermittent centrifugal culture and dynamic bioreactor culture were double than the static culture on the 7th day of cultivation, but there was not significant difference between the intermittent centrifugal culture and dynamic bioreactor culture.

On day 14, the depth of BMSCs infiltrated into the scaffolds were 24.53 ± 6.06 μm, 42.53 ± 13.07 μm, and 115.13 ± 25.44 μm (Fig. 6D–F). There were significant differences between all groups (*P* < 0.05). The infiltration depth of the intermittent centrifugal culture was nearly double the infiltration depth of the static culture. Meanwhile, the infiltration depth of the dynamic bioreactor culture was nearly three times the infiltration depth of the intermittent centrifugal culture.

## Discussion

For regeneration of the AF using a tissue engineering approach, regardless of the scaffold material employed or its specific biomechanical properties, the distribution of sufficient numbers of cells throughout the scaffold is critical, so it is important to improve this so that the cells can infiltrate into the inner part of the scaffolds. In the present study, three culture strategies for BMSCs seeded within electrospinning nanofibrous scaffolds were compared to identify the approach that best promoted BMSCs' infiltration into the scaffolds. The results showed that the dynamic bioreactor culture system best facilitated BMSCs' infiltration into the scaffolds, with a relatively uniform distribution of BMSCs observed within the AYS scaffolds to an average depth of 115.13 ± 25.44 μm after 14 days. This depth corresponded to the vertical distance from the surface of the scaffolds to the deepest point that the BMSCs infiltrated.

Static culture, as used most commonly for monolayer cell culture, was the first culture strategy used in tissue engineering studies and continues to be the most commonly employed technology for the culture of cells within tissue engineering scaffolds. However, with this culture technique, cells typically distribute over the surface of the scaffold, with little cause to infiltrate the scaffold extensively, especially if the pore size is relatively small, as is the case for the AYS nanofibrous scaffolds used in the present study. Greater infiltration can occur with larger pore sizes, but increased porosity corresponds to reductions in the mechanical strength of the scaffold material. Thus, to achieve better distribution of cells throughout the scaffold, a culture approach is needed that promotes the movement of cells within the scaffold pores as well as the movement of medium for nutrient delivery and waste removal.

Previous studies have applied intermittent centrifugal culture to successfully promote the infiltration of the cells within scaffolds^16^, ^17^, including bone marrow stromal cells^18^ and meniscal fibrochondrocytes^19^. Advantages of this technology include the ease of performance under aseptic conditions and the flexibility with regard to scaffold shape and size. However, the distribution of the cells may be uneven due to the directionality of the force applied to the constructs during intermittent centrifugal. In the current study, more cells were observed in the deeper part of the scaffolds with centrifugation than in static culture, but the distribution of the cells was extraordinarily uneven as nearly no cells remained halfway between the deepest penetration depth and the surface. This phenomenon may be associated with the strong force produced during the course of centrifugation. This uneven distribution certainly could affect the subsequent proliferation and differentiation of the cells.

Recently, the method of dynamic bioreactor culture was developed to provide continuous nutrient delivery and metabolic waste removal from tissue engineering constructs. In addition, bioreactors have been designed to generate multiple conditions in order to promote cellular proliferation and differentiation, such as periodic loading via stretching or compression, electric‐pulse stimulation, and intermittent hypoxia^23^, ^24^, ^25^. In the present study, the BioDynamic 5100 bioreactor was employed for the culture of BMSCs within AYS nanofibrous scaffolds. This bioreactor is a type of perfusion bioreactor that can simulate *in vivo* conditions within three‐dimensional constructs. Nutrients and oxygen are delivered to within the central area of the scaffolds by the perfusion flow of the medium, and metabolites are removed simultaneously. This flow is achieved even without the force produced by centrifugation.

With these advantages, the bioreactor has been applied in several studies and achieved desired results. Volkmer *et al*.^26^ used the perfusion bioreactor to culture seeded preosteoblasts on scaffolds for bone tissue engineering and concluded that bioreactor culture prevented cell death in the central part of the scaffolds by maintaining a relatively higher concentration of oxygen than that achieved in static culture. A tubular perfusion system could also be designed for adaptation for more complicated sizes of scaffolds as needed for high volume engineered bone tissue^27^. The advantage of more effective medium and gas exchange makes this method suitable for the current study, because the AYS scaffold must have a relatively small porosity in order to maintain the required mechanical strength. BMSCs seeded onto the AYS scaffolds and cultured using this method were found to infiltrate deeper into the interior region of the scaffolds than with the other two culture methods. These results indicate that dynamic bioreactor culture is a preferable method for distributing and culturing cells within tissue engineering scaffolds.

The current study has some limitations to consider. Only one type of scaffold in one shape and one relatively small size was tested. Thus, more diverse scaffolds of differing material, size, and shape should be examined in future studies. Also, in this study, the scaffolds were placed randomly in the bioreactor, and whether the scaffold position within the bioreactor is associated with the observed effects should be investigated furthermore. Lastly, the differentiation of the BMSCs were not discussed in the current study. Future experiments should address these questions.

The current study compared the ability of three culture methods to promote the infiltration of BMSCs within the interior regions of electrospinning nanofibrous scaffolds intended for use in the tissue engineering‐based repair of the AF. The results showed that dynamic bioreactor culture promoted deeper infiltration of BMSCs into the scaffolds than did static culture or intermittent centrifugal culture. Therefore, dynamic bioreactor culture may be a preferred method for tissue engineering approaches involving scaffolds with a low porosity, such as those needed for repair of the AF.
